# Alcohol Dehydrogenase-1B (rs1229984) and Aldehyde Dehydrogenase-2 (rs671) Genotypes Are Strong Determinants of the Serum Triglyceride and Cholesterol Levels of Japanese Alcoholic Men

**DOI:** 10.1371/journal.pone.0133460

**Published:** 2015-08-18

**Authors:** Akira Yokoyama, Tetsuji Yokoyama, Toshifumi Matsui, Takeshi Mizukami, Mitsuru Kimura, Sachio Matsushita, Susumu Higuchi, Katsuya Maruyama

**Affiliations:** 1 National Hospital Organization Kurihama Medical and Addiction Center, Kanagawa, 239–0841, Japan; 2 Department of Health Promotion, National Institute of Public Health, Saitama, 351–0104, Japan; 3 Department of Geriatric Medicine, Kyorin University Hospital, Tokyo, 181–8611, Japan; Harvard Medical School, UNITED STATES

## Abstract

**Background:**

Elevated serum triglyceride (TG) and high-density-lipoprotein cholesterol (HDL-C) levels are common in drinkers. The fast-metabolizing alcohol dehydrogenase-1B encoded by the ADH1B*2 allele (vs. ADH1B*1/*1 genotype) and inactive aldehyde dehydrogenase-2 encoded by the ALDH2*2 allele (vs. ALDH2*1/*1 genotype) modify ethanol metabolism and are prevalent (≈90% and ≈40%, respectively) in East Asians. We attempted to evaluate the associations between the ADH1B and ALDH2 genotypes and lipid levels in alcoholics.

**Methods:**

The population consisted of 1806 Japanese alcoholic men (≥40 years) who had undergone ADH1B and ALDH2 genotyping and whose serum TG, total cholesterol, and HDL-C levels in the fasting state had been measured within 3 days after admission.

**Results:**

High serum levels of TG (≥150 mg/dl), HDL-C (>80 mg/dl), and low-density-lipoprotein cholesterol (LDL-C calculated by the Friedewald formula ≥140 mg/dl) were observed in 24.3%, 16.8%, and 15.6%, respectively, of the subjects. Diabetes, cirrhosis, smoking, and body mass index (BMI) affected the serum lipid levels. Multivariate analysis revealed that the presence of the ADH1B*2 allele and the active ALDH2*1/*1 genotype increased the odds ratio (OR; 95% confidence interval) for a high TG level (2.22 [1.67–2.94] and 1.39 [0.99–1.96], respectively), and decreased the OR for a high HDL-C level (0.37 [0.28–0.49] and 0.51 [0.37–0.69], respectively). The presence of the ADH1B*2 allele decreased the OR for a high LDL-C level (0.60 [0.45–0.80]). The ADH1B*2 plus ALDH2*1/*1 combination yielded the highest ORs for high TG levels and lowest OR for a high HDL-C level. The genotype effects were more prominent in relation to the higher levels of TG (≥220 mg/dl) and HDL-C (≥100 mg/dl).

**Conclusions:**

The fast-metabolizing ADH1B and active ALDH2, and especially a combination of the two were strongly associated with higher serum TG levels and lower serum HDL-C levels of alcoholics. The fast-metabolizing ADH1B was associated with lower serum LDL-C levels.

## Introduction

Fast-metabolizing alcohol dehydrogenase-1B (ADH1B, rs1229984), which is encoded by the ADH1B*2 allele (vs. ADH1B*1/*1 genotype) and inactive aldehyde dehydrogenase-2 (ALDH2; rs671), which is encoded by the ALDH2*2 allele (vs. ALDH2*1/*1 genotype) have an inhibitory effect on alcohol drinking and are prevalent (e.g., ≈90% and ≈40%, respectively) in East Asians but are uncommon (e.g., ≈10% and ≈0%, respectively) among individuals of European and African descent [1,2]. Most homozygous inactive ALDH2*2⁄*2 carriers are nondrinkers because they experience severe acetaldehydemia and unpleasant flushing responses if they drink alcohol. Other combinations of the ADH1B and ALDH2 genotypes have been shown to modify ethanol and acetaldehyde metabolism during ethanol intoxication [3,4] and the susceptibility to various physical comorbidities among Japanese alcoholics [5–7].

Excessive alcohol drinking increases the risk of stroke, especially hemorrhagic stroke [8–10]. A meta-analysis of 22 prospective studies indicated that long-term alcohol consumption was more strongly associated with a risk of stroke in Asian populations than in Western populations [11]. The ADH1B and ALDH2 gene polymorphisms in Japanese alcoholics modify cardiovascular risk factors such as weight gain [12], hypertension [6], and diabetes mellitus [6]; therefore, these polymorphisms may play an indirect role in the development of cardiovascular diseases.

It has long been known that alcohol consumption is positively associated with high serum triglyceride (TG) and high-density-lipoprotein cholesterol (HDL-C) levels [13], and negatively associated with serum low-density lipoprotein cholesterol (LDL-C) levels [14]. The alcohol-induced increase in serum HDL-C levels contributes to the prevention of coronary heart disease [15], while a meta-analysis of 23 prospective studies has shown that the total cholesterol levels and LDL-C levels were inversely associated with the risk of hemorrhagic stroke [16]. Alcohol-induced alterations in the serum lipid profile are reportedly affected by ADH1B and ALDH2 gene polymorphisms. A small study of Japanese moderate-to-heavy drinkers demonstrated that the fast-metabolizing ADH1B*2/*2 genotype, but not the ALDH2 genotype, was associated with high serum TG levels [17]. A genome-wide association study in Chinese men showed that the active ALDH2*1/*1 genotype, not the ADH1B genotype, further increased the alcohol-induced increase in serum TG levels [18], and a meta-analysis of seven East Asian populations revealed higher serum HDL-C levels among active ALDH2*1/*1 homozygotes [19]. A meta-analysis of 56 studies examining subjects of European descent demonstrated the presence of better cardiovascular markers (including low levels of serum non-HDL-C, body mass index [BMI], and blood pressure) and lower risks of coronary heart disease and ischemic stroke among drinkers with the ADH1B*2 allele, compared with drinkers with the ADH1B*1/*1 genotype [20]. Thus, the associations between the lipid profile and the ADH1B and ALDH2 genotypes in drinkers may affect their risk of cardiovascular events.

In the present study we investigated the effects of the ADH1B and ALDH2 genotypes, drinking habits, smoking habits, BMI, and physical comorbidities on the serum lipid levels of Japanese alcoholic men.

## Materials and Methods

### Subjects

The population of this study consisted of 1806 Japanese alcoholic men 40 years of age or over who met all of the following criteria: (a) admitted to the Kurihama Medical and Addiction Center for treatment of alcohol dependence for the first time between May 2004 and December 2011, (b) was evaluated for the presence of physical comorbidities, (c) underwent ADH1B and ALDH2 genotyping, (d) and underwent blood tests for the serum TG, total cholesterol, and HDL-C levels early in the morning after overnight fast within 3 days after admission. The LDL-C levels of 1763 subjects whose fasting TG levels were below 400 mg/dl were calculated by the Friedewald formula [21]. The blood chemistry examination was performed using a Toshiba TBA-120FR chemistry analyzer (Toshiba Medical Systems Corporation, Tochigi, Japan). The ethics committee of the Center reviewed and approved the proposed study, and each of the participants gave his written informed consent.

All of the alcoholics who participated in this study met the DSM-IV criteria for alcohol dependence [22]. We asked each participant when he was in a sober state about the usual amount(s) and type(s) of alcoholic beverage(s) he consumed during the preceding year and the number of cigarettes a day he currently smoked. Usual alcohol consumption was expressed in grams of ethanol per day calculated by using standard conversion factors for alcoholic beverages. Beer and low-malt beer were assumed to be 5% ethanol (v/v); wine, 12%; sake, 16%; shochu, 25%; and whiskey, 40%.

Dyslipidemia was defined according to the guidelines of the Japan Atherosclerosis Society (TG ≥150 mg/dl, HDL-C <40 mg/dl, HDL-C >80 mg/dl, and LDL-C ≥140 mg/dl [23]). Subjects received a routine examination that included a physical examination, blood tests, chest and abdominal X-ray examinations, upper gastrointestinal endoscopy, abdominal ultrasound examination, and abdominal computed tomography examination. The clinical diagnoses of comorbidities were made after alcohol detoxification. The diagnosis of liver cirrhosis was made on the basis of the results of the physical examination, blood tests, and imaging studies or the detection of esophagogastric varices. Diabetes mellitus was recorded as present if the fasting blood glucose level was 126 mg/dl or more, the nonfasting blood glucose level was 200 mg/dl or more, or the subject was currently taking medication for diabetes mellitus.

### ADH1B and ALDH2 genotyping

Polymerase chain reaction-restriction fragment length polymorphism (PCR-RFLP) methods were performed on lymphocyte DNA samples to genotype ADH1B and ALDH2 [3].

### Statistical analysis

Values were expressed as means and standard deviation (SD) or standard error (SE), or percentage values. Analysis of covariance (ANCOVA) or Cochran-Mantel-Haenszel test was used to compare mean values or proportions between groups after adjusted for age, respectively. Normality of data was checked using a histogram. The non-parametric van Elteren test was used to compare non-normally distributed data between groups after adjusted for age. Multiple linear regression analysis or multiple logistic regression analysis was used to test for independent relationships of lipid levels with other factors. Since TG levels were strongly skewed to the right, the logarithmically transformed values were used for the analyses. The estimated regression coefficients for log-transformed TG levels by the multiple linear regression model were back-transformed to express the relative difference in TG levels according to the dependent variables. The measure of central tendency for TG was expressed as a geometric least square mean and a geometric standard error. A multiple logistic regression model was used to assess the interaction effects of ADH1B genotype (*1/*1 or *2 carrier) and ALDH2 genotype (*1/*1 or *1/*2) on lipid values; odds ratios were estimated using the combinatorial group of ADH1B*1/*1 and ALDH2*1/*2 as the reference; a p-value for homogeneity was calculated to test the overall null hypothesis for the combinatorial genotype groups; a p-value for interaction of ADH1B and ALDH2 genotypes was calculated to assess whether the effect of ADH1B on lipid values is modified by ALDH2, or vice versa. P values <0.05 were considered evidence of statistical significance. All statistical analyses were performed by using the SAS software program (version 9.2; SAS Institute, Cary, NC).

## Results

High serum levels of TG (≥150 mg/dl), HDL-C (>80 mg/dl), and LDL-C (≥140 mg/dl) and low serum levels of HDL-C (<40 mg/dl) were observed in 24.3%, 16.8%, 15.6%, and 18.1%, respectively, of the subjects. Table 1 shows the background factors of the subjects. Significantly younger age was observed in the groups with the high TG and LDL-C levels, and all the subsequent analyses were performed with age-adjustment. Significantly less alcohol consumption and more cigarette smoking was observed in the high TG level group. BMI was significantly higher in the groups with high TG and LDL-C levels, and significantly lower in the group with high HDL-C levels. Frequency of cirrhosis was significantly lower in the groups with high TG, HDL-C, and LDL-C levels. Frequency of diabetes was significantly higher in the high TG level group and significantly lower in the high HDL-C level group.

**Table 1:** Background of Japanese alcoholic men according to whether they had abnormal serum lipid values.

		Serum TG level ≥150 mg/dl		age- adjusted	Serum HDL-C level >80 mg/dl		age- adjusted	Serum LDL-C level ≥140 mg/dl		age- adjusted
	Total	Present	Absent	p	Present	Absent	p	Present	Absent	p
Number of subjects		438	1368		304	1502		267	1496	
Age (years)										
Mean±SD	56.2±9.8	53.3±8.8	57.1±9.9	< .0001	56.1±10.0	56.2±9.7	0.83	54.5±9.3	56.6±9.8	0.001
Usual alcohol intake (g ethanol/day)										
Mean±SE	121±2	119±4	121±2	0.034	124±4	120±2	0.43	126±5	119±2	0.60
Alcoholic beverage most frequently consumed										
Beer / low-malt beer	13.3%	14.7%	12.9%		11.9%	13.6%		15.7%	12.5%	
Sake	20.2%	17.7%	21.0%		17.5%	20.7%		15.7%	20.9%	
Shochu	56.2%	59.9%	55.1%		56.1%	56.3%		61.0%	55.9%	
Whiskey	9.2%	6.4%	10.0%		12.9%	8.4%		6.7%	9.6%	
Wine	1.1%	1.4%	1.0%	0.19	1.7%	1.0%	0.075	0.7%	1.1%	0.18
Cigarette smoking										
Non-smokers	23.3%	17.1%	25.3%		24.3%	23.1%		24.3%	23.5%	
1–19 cigarettes/day	23.9%	22.4%	24.3%		21.1%	24.4%		19.9%	24.4%	
≥20 cigarettes/day	52.8%	60.5%	50.4%	0.011	54.6%	52.5%	0.86	55.8%	52.1%	0.78
Cigarettes/day (mean±SE)	16.5±0.3	19.2±0.6	15.6±0.3	0.0002	16.4±0.8	16.5±0.3	0.97	16.7±0.8	16.3±0.3	0.89
Body mass index (kg/m^2^)										
Mean±SE	21.6±0.8	22.2±0.2	21.4±0.1	0.001	20.9±0.2	21.7±0.1	0.0001	22.6±0.2	21.4±0.1	< .0001
Liver cirrhosis										
Present	18.7%	14.2%	20.2%		7.2%	21.0%		12.4%	19.7%	
Absent	81.3%	85.8%	79.8%	0.002	92.8%	79.0%	< .0001	87.6%	80.3%	0.003
Diabetes mellitus										
Present	20.0%	25.6%	18.2%		14.5%	21.1%		19.5%	19.7%	
Absent	80.0%	74.4%	81.8%	< .0001	85.5%	78.9%	0.009	80.5%	80.3%	0.87
Hypertension being treated with medication										
Present	26.3%	26.7%	26.2%		24.7%	26.6%		26.6%	26.2%	
Absent	73.7%	73.3%	73.8%	0.090	75.3%	73.4%	0.49	73.4%	73.8%	0.42

TG, triglyceride; HDL-C, high-density-lipoprotein cholesterol; LDL-C, low-density-lipoprotein cholesterol; SD, standard deviation; SE, standard error. P values for categorical data were adjusted for age by Cochran-Mantel-Haenszel test for trend for cigarette smoking and ketonuria; for homogeneity for other variables. P values for means were adjusted for age by non-parametric van Elteren test (for cigarettes/day) or analysis of covariance (ANCOVA, for other variables).

The TG levels (log-transformed values) were correlated with the HDL-C levels (Pearson’s correlation coefficient (*r*) = -0.273, p<0.0001) and LDL-C levels (*r* = 0.197, p<0.0001). Table 2 shows the serum TG, HDL-C, and LDL-C levels of the subjects according to their ADH1B and ALDH2 genotypes. It is well known that the alcohol drinking habits are strongly affected by the genotypes of ALDH2 and ADH1B and the genotype distributions in alcoholics do not follow the Hardy-Weinberg equilibrium [2]. As expectedly, the genotype distributions of ALDH2 and ADH1B in the present study were significantly deviated from Hardy-Weinberg equilibrium (ALDH2: χ^2^ = 12.6, df = 1, p<0.001; ADH1B: χ^2^ = 200.5, df = 1, p<0.001) and the frequency of ALDH2*2/*2 was zero. Current alcohol consumption did not differ according to the ADH1B and ALDH2 genotypes. The fast-metabolizing ADH1B*2 allele carriers had significantly higher TG levels and significantly lower HDL-C and LDL-C levels. The active ALDH2*1/*1 carriers had significantly higher TG levels and significantly lower HDL-C levels.

**Table 2:** Comparisons between the serum lipid levels of Japanese alcoholic men according to their ADH1B and ALDH2 genotypes.

		ADH1B genotype			Adjusted	ALDH2 genotype		Adjusted
	Total	*1/*1	*1/*2	*2/*2	p ^b^	*1/*1	*1/*2	p ^b^
Number of subjects	1806	490	592	724		1528	278	
Usual alcohol intake (g ethanol/day)								
Adjusted LSM±SE ^a^	121±2	125±4	123±3	116±3	0.12	120±2	122±5	0.79
Serum TG level								
≥250 mg/dl	6.7%	4.3%	7.9%	7.3%		7.2%	4.0%	
150–249 mg/dl	17.6%	13.9%	18.6%	19.2%		18.1%	14.7%	
<150 mg/dl	75.7%	81.8%	73.5%	73.5%	< .0001	74.7%	81.3%	0.020
Adjusted GLSM, GSE ^a^	111.5, 1.01	99.9, 1.02	117.0, 1.02	115.5, 1.02	< .0001	113.2, 1.01	102.8, 1.03	0.005
Serum HDL level								
≥100 mg/dl	5.9%	12.9%	4.1%	2.6%		5.0%	10.4%	
81–99 mg/dl	11.0%	13.9%	11.1%	8.8%		9.8%	17.3%	
40–80 mg/dl	65.1%	59.8%	64.7%	69.1%		66.2%	59.4%	
<40 mg/dl	18.1%	13.5%	20.1%	19.5%	< .0001	19.0%	12.9%	< .0001
Adjusted LSM±SE ^a^	59.1±0.6	67.6±1.1	56.2±1.0	55.7±0.9	< .0001	57.7±0.6	66.6±1.5	< .0001
Serum LDL-C level	n = 1763	n = 479	n = 576	n = 708		n = 1488	n = 275	
≥140 mg/dl	15.1%	22.3%	13.7%	11.4%		15.4%	13.8%	
<140 mg/dl	84.9%	77.7%	86.3%	88.6%	< .0001	84.6%	86.2%	0.53
Adjusted LSM±SE ^a^	101.0±0.9	113.5±1.7	97.6±1.6	95.2±1.4	< .0001	101.1±1.0	100.4±2.3	0.78

TG, triglyceride; HDL-C, high-density-lipoprotein cholesterol; LDL-C, low-density-lipoprotein cholesterol.

^a^ LSM, least square mean adjusted for age and usual alcohol intake by analysis of covariance (ANCOVA); SE, standard error; GLSM, geometric LSM; GSE, geometric SE.

^b^ P for trend adjusted for age and usual alcohol intake by ANCOVA (for mean values), logistic regression model (for LDL-C category), or proportional odds model (for TG and HDL categories).

Determinants of the serum TG, HDL-C, and LDL-C levels were identified by a multiple linear regression analysis (Table 3). TG levels were 22.3% higher and 7.8% higher, respectively, in the group with the ADH1B*2 allele (vs. the ADH1B*1/*1 genotype) and group with the ALDH2*1/*1 genotype (vs. the ALDH2*1/*2 genotype), and HDL-C levels were 11.77 mg/dl lower and 7.02 mg/dl lower, respectively, and all of the differences were significant. LDL-C levels were 13.45 mg/dl lower in the group with the ADH1B*2 allele (vs. the ADH1B*1/*1 genotype).

**Table 3:** Multiple linear regression analysis to predict serum lipid levels of Japanese alcoholic men.

	TG levels (mg/dl)			HDL-C levels (mg/dl)			LDL-C levels (mg/dl)		
Independent variables	Regression coefficient (relative difference)^b^	SE	p	Regression coefficient	SE	p	Regression coefficient	SE	p
ADH1B genotype *2 carrier vs. *1/*1	22.3%	2.8%	< .0001	-11.77	1.26	< .0001	-13.45	2.03	< .0001
ALDH2 genotype *1/*1 vs. *1/*2	7.8%	3.4%	0.024	-7.02	1.49	< .0001	2.08	2.41	0.39
Age, per +10 years	-9.6%	1.4%	< .0001	-0.92	0.61	0.13	-1.44	0.99	0.15
Usual ethanol consumption, per +22g/day	-1.1%	0.3%	0.001	0.18	0.15	0.23	-0.27	0.25	0.28
Alcoholic beverage most frequently consumed									
Beer / low-malt beer	referent		0.77^a^	referent		0.71^a^	referent		0.24^a^
Sake	-1.4%	4.4%	0.74	0.49	1.93	0.80	-4.09	3.15	0.19
Shochu	-2.2%	3.7%	0.55	0.87	1.65	0.60	-0.02	2.70	0.99
Whiskey	-5.9%	5.3%	0.24	3.07	2.32	0.19	-5.15	3.77	0.17
Wine	4.2%	12.5%	0.73	3.11	5.30	0.56	3.83	8.94	0.67
Current smoking, per +10 cigarettes	4.7%	1.0%	< .0001	-0.85	0.43	0.048	-0.21	0.69	0.77
BMI, per +1.0 kg/m^2^	2.8%	0.4%	< .0001	-1.06	0.17	< .0001	1.85	0.27	< .0001
Presence of diabetes vs. absence	13.6%	3.1%	< .0001	-1.26	1.38	0.36	-2.34	2.25	0.30
Presence of hypertension vs. absence	4.1%	2.9%	0.16	3.22	1.28	0.012	-0.06	2.08	0.98
Presence of liver cirrhosis vs. absence	-21.0%	3.2%	< .0001	-17.08	1.42	< .0001	-16.68	2.31	< .0001

^a^ P for homogeneity among the alcohol beverages.

^b^ Since TG levels distributed log-normally, multiple linear regression analysis was done for the log-transformed TG value and the coefficients were shown in terms of relative difference (e.g., TG levels were 22.3% higher in ADH1B*2 carrier as compared to ADH1B*1/*1).

Higher age was significantly associated with lower TG levels. Higher numbers of cigarettes smoked were significantly associated with higher TG levels and lower HDL-C levels. Higher BMI was significantly associated with higher TG and LDL-C levels and with lower HDL-C levels. The presence of diabetes was significantly associated with higher TG levels. The presence of hypertension was significantly associated with higher HDL-C levels. The presence of cirrhosis was significantly associated with lower TG, HDL-C, and LDL-C levels.

Multiple logistic regression analysis that included the same independent variables as those included in the multiple linear regression analysis presented in Table 3 showed that the presence of the ADH1B*2 allele and the ALDH2*1/*1 genotype increased the odds ratio [OR, 95% confidence interval (CI)] for a TG level ≥150 mg/dl (2.22 [1.67–2.94] and 1.39 [0.99–1.96], respectively), and the OR (95%CI) for a TG level ≥250 mg/dl (3.19 [1.88–5.43] and 2.02 [1.03–3.98], respectively, Table 4).

**Table 4:** Multiple logistic regression analysis of dyslipemia in Japanese alcoholic men according to their ADH1B and ALDH2 genotypes after adjustment for other independent variables.

Genotype (independent variables)	TG level ≥150 mg/dl, vs. <150 mg/dl			TG level ≥250 mg/dl, vs. <150 mg/dl			HDL-C level >80 mg/dl, vs. ≤80 mg		
	OR	95% CI	p	OR	95% CI	p	OR	95% CI	p
ADH1B *2 allele vs. *1/*1	2.22	(1.67–2.94)	< .0001	3.19	(1.88–5.43)	< .0001	0.37	(0.28–0.49)	< .0001
ALDH2 *1/*1 vs. *1/*2	1.39	(0.99–1.96)	0.057	2.02	(1.03–3.98)	0.042	0.51	(0.37–0.69)	< .0001
Genotype (independent variables)	HDL-C level ≥100 mg/dl, vs. ≤80 mg/dl			HDL-C level <40 mg/dl, vs. ≥40 mg/dl			LDL-C level ≥140 mg/dl, vs. <140 mg/dl		
	OR	95% CI	p	OR	95% CI	p	OR	95% CI	p
ADH1B *2 allele vs. *1/*1	0.18	(0.12–0.29)	< .0001	1.54	(1.12–2.14)	0.009	0.60	(0.45–0.80)	0.001
ALDH2 *1/*1 vs. *1/*2	0.48	(0.30–0.78)	0.003	1.40	(0.94–2.08)	0.101	1.16	(0.79–1.70)	0.441

OR, odds ratio; CI, confidence interval.

ADH1B and ALDH2 genotypes were simultaneously entered into a model adjusted for age, usual ethanol consumption, alcoholic beverage most frequently consumed, current smoking, BMI, presence of diabetes, hypertension, and liver cirrhosis.

The presence of the ADH1B*2 allele and ALDH2*1/*1 genotype decreased the OR (95%CI) for an HDL-C level >80 mg/dl (0.37 [0.28–0.49] and 0.51 [0.37–0.69], respectively) and the OR (95%CI) for an HDL-C level ≥100 mg/dl (0.18 [0.12–0.29] and 0.48 [0.30–0.78], respectively). The presence of the ADH1B*2 allele increased the OR (95%CI) for an HDL-C level <40 mg/dl (1.54 [1.12–2.14]) and decreased the OR (95%CI) for an LDL-C level ≥140 mg/dl (0.60 [0.45–0.80]).

Table 5 shows the associations between the serum TG, HDL-C, and LDL-C levels and the ADH1B and ALDH2 genotype combinations after adjusting for the same independent variables as in Table 3. The ADH1B*2 allele plus ALDH2*1/*1 genotype combination had a significant and the strongest effect on TG levels ≥150 mg/dl and ≥250 mg/dl (OR [95%CI] = 2.29 [1.25–4.18] and 9.63 [1.29–72.09], respectively), and HDL-C levels >80 mg/dl and ≥100 mg/dl (0.26 [0.16–0.43] and 0.11 [0.05–0.22], respectively).

**Table 5:** Multiple logistic regression analysis of dyslipemia in Japanese alcoholic men according to combinations of ADH1B and ALDH2 genotypes after adjustment for other independent variables.

ADH1B genotype & ALDH2 genotype	TG level ≥150 mg/dl, vs. <150 mg/dl			TG level ≥250 mg/dl, vs. <150 mg/dl			HDL-C level >80 mg/dl, vs. ≤80 mg		
	OR	95% CI	p	OR	95% CI	p	OR	95% CI	p
*1/*1 & *1/*2 (n = 90)	1	(referent)		1	(referent)		1	(referent)	
*1/*1 & *1/*1 (n = 400)	0.95	(0.50–1.82)	0.89	3.13	(0.40–24.40)	0.28	0.87	(0.52–1.46)	0.60
*2 allele & *1/*2 (n = 188)	1.44	(0.72–2.89)	0.30	5.09	(0.62–42.06)	0.13	0.72	(0.40–1.27)	0.25
*2 allele & *1/*1 (n = 1128)	2.29	(1.25–4.18)	0.007	9.63	(1.29–72.09)	0.027	0.26	(0.16–0.43)	< .0001
P for homogeneity ^a^			< .0001			0.0001			< .0001
P for interaction ^b^			0.19			0.65			0.009
ADH1B genotype & ALDH2 genotype	HDL-C level ≥100 mg/dl, vs. ≤80 mg/dl			HDL-C level <40 mg/dl, vs. ≥40 mg/dl			LDL-C level ≥140 mg/dl, vs. <140 mg/dl		
	OR	95% CI	p	OR	95% CI	p	OR	95% CI	p
*1/*1 & *1/*2	1	(referent)		1	(referent)		1	(referent)	
*1/*1 & *1/*1	0.74	(0.38–1.46)	0.38	0.88	(0.43–1.79)	0.72	1.88	(0.97–3.66)	0.061
*2 allele & *1/*2	0.36	(0.16–0.82)	0.015	0.90	(0.41–1.97)	0.78	1.15	(0.55–2.43)	0.71
*2 allele & *1/*1	0.11	(0.05–0.22)	< .0001	1.50	(0.78–2.91)	0.23	1.01	(0.53–1.93)	0.97
P for homogeneity ^a^			< .0001			0.007			0.001
P for interaction ^b^			0.061			0.14			0.063

OR, odds ratio; CI, confidence interval.

ORs were adjusted for age, usual ethanol consumption, alcoholic beverage most frequently consumed, current smoking, BMI, presence of diabetes, hypertension, and liver cirrhosis.

^a^ P for overall no-association between the 4 combinatorial groups of ADH1B and ALDH2 genotypes.

^b^ P for interaction of ADH1B by ALDH2 genotypes; a significant p value means that the effect of ADH1B on lipid values is modified by ALDH2, or vice versa

## Discussion

This cross-sectional study of Japanese alcoholic men demonstrated that the fast-metabolizing ADH1B and active ALDH2 encoded by the ADH1B*2 allele and ALDH2*1/*1 genotype, respectively, and especially a combination of the two had significant increasing effects on serum TG levels and decreasing effects on serum HDL-C levels. The ADH1B*2 allele was also associated with lower serum LDL-C levels. Since current alcohol consumption by the alcoholics did not differ according to the ADH1B and ALDH2 genotypes, these effects may be attributable to faster ethanol and acetaldehyde metabolism because of the ADH1B*2 allele and the ALDH2*1/*1 genotype.

**Fig 1:**
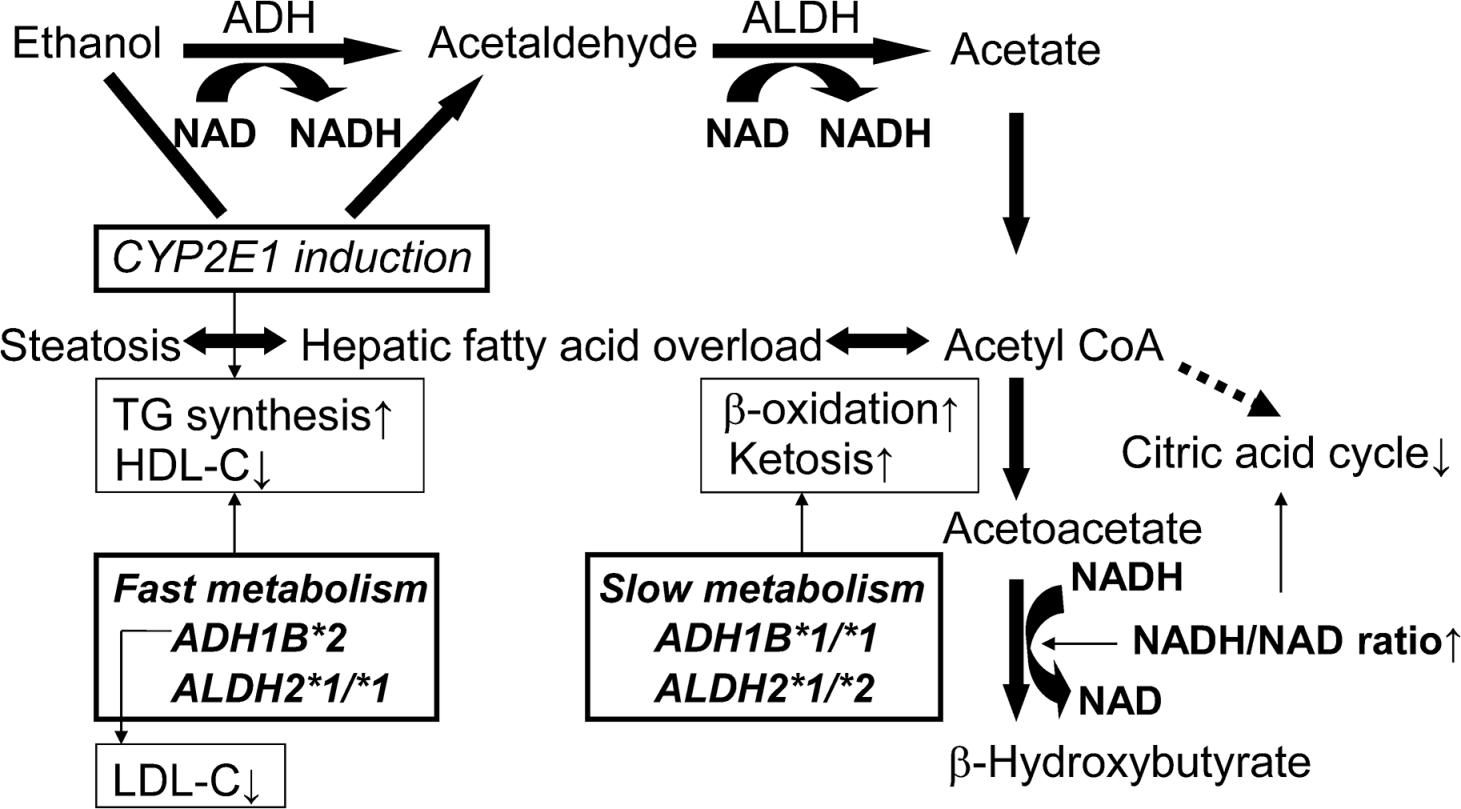
Interactions between the metabolism of ethanol and lipids and alcohol-metabolizing enzymes in alcoholics. ADH, alcohol dehydrogenase; ALDH, aldehyde dehydrogenase; CYP2E1, cytochrome p4502E1; HDL-C, high-density lipoprotein cholesterol; LDL-C, low-density lipoprotein cholesterol; TG, triglyceride. Thin arrows indicate a promoting effect.

Dietary ethanol has been demonstrated to increase postprandial hypertriglyceridemia [24]. High blood ethanol levels directly stimulate lipolysis in adipose tissue [13]. TG synthesis and mitochondrial -oxidation/ketogenesis are two major pathways by which fatty acids in fatty-acid-overloaded hepatocytes are removed (Fig 1). The increase in NADH/NAD ratio that occurs during ethanol metabolism and the shortage of citrate cycle intermediates due to nutritional deficiencies inhibit the activity of the citric acid cycle, which results in relative suppression of mitochondrial β-oxidation. The results of our most recent study demonstrated that the presence of the ADH1B*2 allele and presence of ALDH2*1/*1 genotype in Japanese alcoholic men were negatively associated with their urinary ketone levels [25]. Thus, faster ethanol and acetaldehyde metabolism because of the ADH1B*2 allele and the ALDH2*1/*1 genotype may play a suppressive role on mitochondrial -oxidation/ketogenesis and increase TG synthesis in the liver of alcoholics. The presence of the ADH1B*2 allele and ALDH2*1/*1 genotype has been shown to be positively associated with the severity of alcoholic liver disease in Japanese alcoholic men [6], and the pro-steatosis effects of the ADH1B*2 allele and ALDH2*1/*1 genotype on fatty acid metabolism may increase susceptibility to alcoholic liver disease.

Ethanol consumption has been shown to down-regulate the activity of peroxisome proliferator-activated receptor α (PPARα) and to up-regulate the activity of sterol regulatory element-binding protein-1c (SREBP-1c), and the alterations of these fatty acid regulators promote TG synthesis and suppress -oxidation in the liver [26]. These effects on PPARα and SREBP-1c may be mediated by ethanol metabolism rather than by ethanol exposure per se, since inhibition of ethanol oxidation by 4-methylpyrazole, an ADH inhibitor, blocked these effects in rat hepatoma cell lines [27,28]. That is compatible with the higher serum TG levels and lower urine ketone levels [25] that have been demonstrated in Japanese alcoholic who had the fast ethanol metabolizing ADH1B.

The microsomal ethanol oxidizing system, including cytochrome P450 2E1 (CYP2E1), is induced by ethanol in daily or heavy drinkers [29]. In a recent study we demonstrated that Japanese alcoholic men with the ADH1B*2 allele had lower BMIs [12], lower blood ethanol levels in the morning after drinking the day before [3,4], and a higher prevalence of advanced alcoholic liver disease [6] than Japanese alcoholic men with the ADH1B*1/*1 genotype did. We have proposed the hypothesis that CYP2E1 activity is more strongly induced by ADH1B*2-associated fast ethanol metabolism in alcoholics, because this hypothesis explains all of the ADH1B-associated differences in BMI, alcohol metabolism, and the prevalence of advanced liver disease [4,6,12]. Strong CYP2E1 induction is associated with ethanol energy expenditure, fast ethanol elimination, and alcohol-induced liver disease [29]. Ethanol metabolism accelerated by ethanol-induced CYP2E1 may also promote TG synthesis, because feeding CYP2E1-knockout mice ethanol resulted in up-regulation of PPARα, and because the mice did not develop fatty liver [30].

After ethanol feeding the ALDH2-knockout mice had lower hepatic SREBP-1c levels and a lower degree of hepatic steatosis than wild-type mice [31], although an *in vitro* study in rat hepatoma cell lines showed that cyanamide, an ALDH inhibitor, increased the effects of ethanol on the up-regulation of SREBP-1c [28]. The results of the present study showed an association between the lower TG levels and the inactive ALDH2*1/*2 genotype, which is a decisive determinant of high acetaldehyde exposure [32]. High ketonuria levels have been detected in a higher proportion of alcoholics with the inactive ALDH2*1/*2 genotype than in alcoholic with the active ALDH2*1/*1 genotype [25]. These observations suggest that high acetaldehyde exposure in ALDH2*1/*2 alcoholics accelerates mitochondrial β-oxidation/ketogenesis and decelerates alcohol-induced increases in serum TG levels.

A protective effect of moderate alcohol consumption against coronary heart disease has been well established, and the alcohol-induced increase in serum HDL-C levels has been postulated as a major mechanism to explain it [15]. Moderate drinking increases the plasma concentrations of the major HDL components (HDL-C, apoA-I and–II) through an increase in the HDL apoprotein transport rate [33]. Slow ethanol and acetaldehyde metabolism may promote the high-HDL-C-level associated changes in alcoholics, because the ADH1B*2 allele and ALDH2*1/*1 genotype, especially in combination, were found to be associated with lower HDL-C levels in this study. Their decreasing effects on HDL-C levels may occur in conjunction with their increasing effects on TG levels, because the HDL-C levels and TG levels were intercorrelated. However, when the log-transformed TG levels were added as an independent variable in the logistic model in Table 4, the OR for a high HDL-C level (>80 mg/dl) of the presence of the ADH1B*2 allele (OR [95%CI] = 0.47 [0.35–0.63], p<0.001) and by ALDH2*1/*1 genotype (0.52 [0.38–0.72], p<0.001) remained strongly significant.

High alcohol consumption has been reported to be inversely associated with serum adiponectin levels in Japanese men with the ADH1B*2/*2 genotype but not with any of the other ADH1B genotypes [34]. Moderate alcohol consumption has been found to be associated with high serum adiponectin levels in non-Asians [35,36], most of whom are ADH1B*1/*1 carriers, but studies of Japanese males showed an inverse association [35,37]. Since decreases in serum adiponectin levels are associated with increases in TG levels and decreases in HDL-C levels [38], the increasing effect of the ADH1B*2 allele on TG levels and decreasing effect on HDL-C levels in alcoholics may be mediated by adiponectin.

The ADH1B*2 allele was also associated with lower LDL-C levels in comparison with the ADH1B*1/*1 genotype. The results of studies on the relationship between alcohol consumption and serum LDL-C levels have been inconsistent [36], but a large cross-sectional Japanese study of 25,689 healthy male workers clearly demonstrated that alcohol consumption resulted in dose-dependent decrease in serum LDL-C [14]. The inconsistency may in part be attributable to the racial difference in frequency of the ADH1B*2 allele [1], and the inverse relationship between alcohol consumption and serum LDL-C levels may be more marked in the fast ethanol metabolizers with the ADH1B*2 allele [20,39]. The decreases in the serum HDL-C and LDL-C levels are related to the increasing severity of liver disease [40], and the presence of liver cirrhosis was found to be a strong determinant of the decreases in HDL-C and LDL-C levels in the present study. The severity of liver disease may at least in part account for the suppressive effects of the ADH1B*2 allele on serum HDL-C and LDL-C levels. The positive association between the ALDH2*1/*2 genotype and HDL-C levels of the alcoholic population in our study was in contrast to the results of a meta-analysis of seven East-Asian populations [19], which showed that higher alcohol consumption in active ALDH2*1/*1 homozygotes may play a major role in increasing HDL-C levels. This discrepancy suggests that the effects of the ALDH2-genotype on HDL-C differ with the level of ethanol exposure.

The presence of diabetes was associated with higher TG levels. Current smoking was associated with higher serum TG levels and lower serum HDL-C levels, and higher BMI was associated with higher serum TG and LDL-C levels and lower serum HDL-C levels. These associations are well-known in the Japanese general male populations [14,41] and were significant in the alcoholic male population after adjustment for the ADH1B and ALDH2 genotypes, although susceptibility to diabetes in Japanese alcoholic men is higher in the presence of the ADH1B*2 allele and ALDH2*1/*1 genotype [6], and BMI is strongly negatively association with the presence of the ADH1B*2 allele [12]. A recent meta-analysis of European descent has also confirmed the negative association between BMI and the presence of the ADH1B*2 allele, and the association was much stronger in heavy drinkers than in the other groups [20]. The meta-analysis demonstrated lower serum non-HDL-C levels and a lower risk of coronary heart disease in drinkers with the ADH1B*2 allele than in drinkers with the ADH1B*1/*1 genotype. It is conceivable that the associations between cardiovascular risk factors including lipid profile, BMI, and diabetes and the ADH1B and ALDH2 genotypes in Japanese alcoholics modify their risk of cardiovascular events.

The present study had several limitations. The first limitation was that it was a cross-sectional survey that inquired about usual alcohol consumption in the past year. We did not observe any effects of usual alcohol consumption on HDL-C and LDL-C, and opposite to what we expected, usual alcohol consumption was slightly but significantly lower in the high TG level group after adjustment for age. These findings may be related to the homogeneity of the study population in regard to the subjects’ high alcohol consumption, and affected by unidentified confounders associated with usual alcohol consumption in the alcoholics. A more recent drinking profile that included the pattern of alcohol consumption, e.g., binge drinking, might have been more informative, but recall bias and the reliability of the responses of alcoholics to be asked about their last drink are always problematic. Another limitation was that nutritional status, including caloric intake, food composition, and physical activity were not investigated, and these factors have a considerable impact on lipid metabolism. Other lipid metabolism parameters, such as apoproteins, subfractions of HDL-C and LDL-C, and adiponectin, were not measured. The results might have been more reliable if these factors had been evaluated simultaneously. The ADH1B and ALDH2 genotyping has been performed for 7.6 years of the study period, and there may have been some batch effects on the genotyping. Since the genotyping has been done independently from whether the subjects had lipid alterations or not, the batch effects were not so great, if any, to influence the results. The effects of ADH1B and ALDH2 genotypes on lipid alterations may be associated with unidentified linkage equilibrium with other gene polymorphisms affecting lipid metabolism. Future research may evaluate such linkages. Generalization of the results obtained in our study based on an investigation of treatment-seeking alcoholic men will require confirmation among various drinking populations, including cases with mild alcoholism, and the evaluation in female alcoholics will be a focus of future research.

In conclusion, diabetes, cirrhosis, smoking, and BMI affected the serum lipid levels of the alcoholic male population in this study. The fast-metabolizing ADH1B and active ALDH2, and especially a combination of the two were strongly associated with higher serum TG levels and lower serum HDL-C levels. The fast-metabolizing ADH1B was associated with lower serum LDL-C levels.
